# Additional coils mitigate elevated defibrillation threshold in right-sided implantable cardioverter defibrillator generator placement: a simulation study

**DOI:** 10.1093/europace/euad146

**Published:** 2023-06-14

**Authors:** Shuang Qian, Sofia Monaci, Caroline Mendonca-Costa, Fernando Campos, Philip Gemmell, Hassan A Zaidi, Ronak Rajani, John Whitaker, Christopher A Rinaldi, Martin J Bishop

**Affiliations:** Department of Biomedical Engineering, School of Imaging Sciences and Biomedical Engineering, Kings College London, 4th North Wing, St Thomas’ Hospital, London SE1 7EH, UK; Department of Biomedical Engineering, School of Imaging Sciences and Biomedical Engineering, Kings College London, 4th North Wing, St Thomas’ Hospital, London SE1 7EH, UK; Department of Biomedical Engineering, School of Imaging Sciences and Biomedical Engineering, Kings College London, 4th North Wing, St Thomas’ Hospital, London SE1 7EH, UK; Department of Biomedical Engineering, School of Imaging Sciences and Biomedical Engineering, Kings College London, 4th North Wing, St Thomas’ Hospital, London SE1 7EH, UK; Department of Biomedical Engineering, School of Imaging Sciences and Biomedical Engineering, Kings College London, 4th North Wing, St Thomas’ Hospital, London SE1 7EH, UK; Department of Biomedical Engineering, School of Imaging Sciences and Biomedical Engineering, Kings College London, 4th North Wing, St Thomas’ Hospital, London SE1 7EH, UK; Department of Cardiology, Guy’s and St Thomas’ Hospital, Westminster Bridge Rd, London SE1 7EH, UK; Department of Biomedical Engineering, School of Imaging Sciences and Biomedical Engineering, Kings College London, 4th North Wing, St Thomas’ Hospital, London SE1 7EH, UK; Department of Cardiology, Guy’s and St Thomas’ Hospital, Westminster Bridge Rd, London SE1 7EH, UK; Department of Biomedical Engineering, School of Imaging Sciences and Biomedical Engineering, Kings College London, 4th North Wing, St Thomas’ Hospital, London SE1 7EH, UK; Department of Cardiology, Guy’s and St Thomas’ Hospital, Westminster Bridge Rd, London SE1 7EH, UK; Department of Biomedical Engineering, School of Imaging Sciences and Biomedical Engineering, Kings College London, 4th North Wing, St Thomas’ Hospital, London SE1 7EH, UK

**Keywords:** Computational modelling, Implantable cardioverter defibrillator, Defibrillation threshold, Right-sided generator, CT imaging

## Abstract

**Aims:**

The standard implantable cardioverter defibrillator (ICD) generator (can) is placed in the left pectoral area; however, in certain circumstances, right-sided cans may be required which may increase defibrillation threshold (DFT) due to suboptimal shock vectors. We aim to quantitatively assess whether the potential increase in DFT of right-sided can configurations may be mitigated by alternate positioning of the right ventricular (RV) shocking coil or adding coils in the superior vena cava (SVC) and coronary sinus (CS).

**Methods and results:**

A cohort of CT-derived torso models was used to assess DFT of ICD configurations with right-sided cans and alternate positioning of RV shock coils. Efficacy changes with additional coils in the SVC and CS were evaluated. A right-sided can with an apical RV shock coil significantly increased DFT compared to a left-sided can [19.5 (16.4, 27.1) J vs. 13.3 (11.7, 19.9) J, *P* < 0.001]. Septal positioning of the RV coil led to a further DFT increase when using a right-sided can [26.7 (18.1, 36.1) J vs. 19.5 (16.4, 27.1) J, *P* < 0.001], but not a left-sided can [12.1 (8.1, 17.6) J vs. 13.3 (11.7, 19.9) J, *P* = 0.099). Defibrillation threshold of a right-sided can with apical or septal coil was reduced the most by adding both SVC and CS coils [19.5 (16.4, 27.1) J vs. 6.6 (3.9, 9.9) J, *P* < 0.001, and 26.7 (18.1, 36.1) J vs. 12.1 (5.7, 13.5) J, *P* < 0.001].

**Conclusion:**

Right-sided, compared to left-sided, can positioning results in a 50% increase in DFT. For right-sided cans, apical shock coil positioning produces a lower DFT than septal positions. Elevated right-sided can DFTs may be mitigated by utilizing additional coils in SVC and CS.

## Graphical Abstract

**Graphical Abstract:**
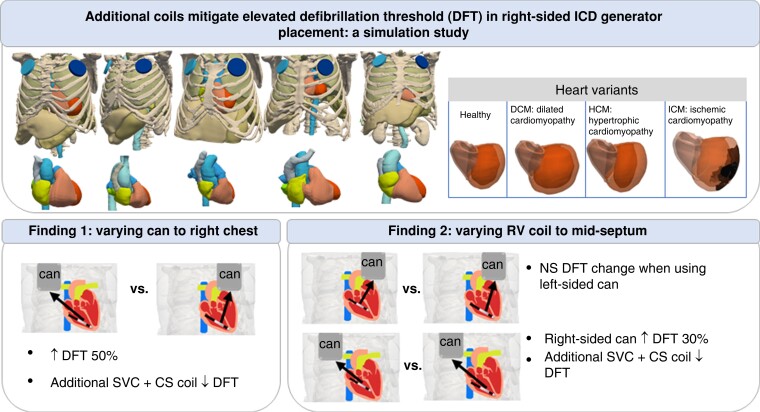
Abbreviations: DFT: defibrillation threshold; SVC: Superior Vena Cava; CS: Coronary Sinus; NS: not significant.

**Translational Perspective**

This computational framework established here can be further expanded to assess the optimization of defibrillation efficacy with various leads/cans placements based on specific anatomical variations such as paediatric or obese patients, along with different cardiac myopathies.

**What’s new?**

Our computational modelling study, based on a large cohort of CT-derived torso models, demonstrated that:

- Using right-sided generators increases defibrillation threshold (DFT) by over 50% compared to left-sided generators.
- The corresponding elevated DFT may be mitigated by utilizing additional coils in the superior vena cava and coronary sinus.
- An apical shock coil positioning is superior (lower DFT) with right-sided generators, compared to the higher septal positions.

## Introduction

The implantable cardioverter defibrillator (ICD) is the most effective life-saving treatment for otherwise lethal cardiac arrhythmias.^1^ Strong biphasic shocks are usually delivered between the active generator (can) and a coil in the right ventricle (RV), spanning the ventricular myocardium. Conventionally, patients will have the can placed in the left pectoral region just under the clavicle and the distal shock lead implanted inside the RV chamber, usually in an apical position. This configuration is not always achievable, due to various conditions or lead-related complications. For example, prior left-sided device infections may require reimplantation to the right pectoral area. There is also an option to place the shocking coil towards the RV mid-septum, rather than the apex as in cardiac resynchronization therapy defibrillator (CRT-D) devices.

In an attempt to improve defibrillation efficacy, an additional coil implanted in the superior vena cava (SVC) either by using a dual-coil system with the second coil within the SVC or a stand-alone SVC coil can also be used.^2^ Several clinical or simulation studies have compared defibrillation efficacy and clinical outcomes of dual-coil with single-coil systems, with inconclusive results, suggesting either no difference^3–5^ or significantly lower defibrillation threshold (DFT).^6,7^ In addition to SVC coils, another coil can also be placed more closely to the left ventricle (LV) through the coronary sinus (CS), similar to the lead placement in CRT-D devices, particularly useful for patients with higher DFTs.^8,9^ Although additional coils may reduce DFT, they may also be associated with increased risks of intervention-related complications, especially when extraction is required.^1,10^ Therefore, such novel configurations are usually treated as backup options for specific cases, with the use of additional coils minimized.

Previous clinical studies have suggested that configurations with right-sided cans have relatively higher DFTs than left-sided, and that SVC coil inclusion in this configuration may thus be beneficial.^11–13^ The effect of various RV coil positions on defibrillation efficacy has also been studied in different experimental setups, with some suggesting apical locations are superior^14,15^ while some suggesting septal locations.^16,17^ However, a controlled comparison of using a right-sided can and/or RV septal coil has not been performed with respect to the conventional RV apical coil and left-sided can configurations, along with the use of additional coils.

Computational models have been widely applied to aid the testing and optimization of ICDs for decades, including important studies seeking to optimize transvenous^15–17^ and subcutaneous^18,19^ configurations, as well as specific implantation strategies for paediatric and congenital patients.^6,20^ Importantly, previous studies have specifically validated the use of realistic electrophysical models to predict the electric potential distribution across the heart’s surface^21^ and whole torso,^22^ allowing the derivation of DFT and impedance that closely replicates clinical measurements.^6,21,23^ In this study, we generated a cohort of CT-derived high-resolution whole torso computational models to assess defibrillation efficacy of commonly necessitated ICD modifications, including right-sided can and RV septal coils, compared with conventional ICD configurations. Additional coils in the SVC and CS were incorporated into the configurations to assess how DFT may be improved in these circumstances.

## Methods

### Model generation

#### Whole torso model generation

Anonymized whole torso CT scans (0.7×0.7×0.5mm) along with additional higher resolution contrast cardiac scans (0.3×0.3×0.5mm) from five patients undergoing trans-catheter aortic valve implantation planning scans were used. All patients consented for the use of their data in ethically approved research: UK Research Ethics Committee: 19/HRA/0502 and 15/LO/1803.

**Figure 1.**
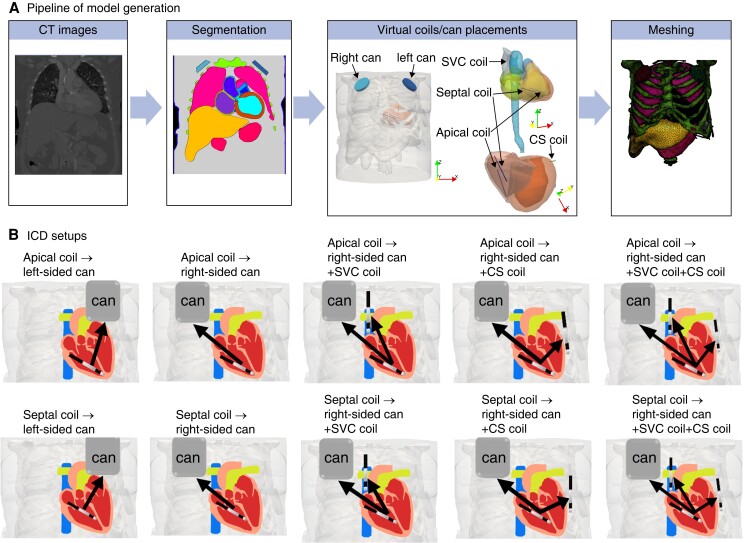
Model generation and ICD setups. (A) Pipeline of generating torso models with embedded detailed cardiac models with commonly used ICD coils/cans. (B) Schematic of ICD setups assessed in this study. The RV apical coil to the left-sided can represents the conventional ICD configuration.

*Figure 1* shows the semi-automatic pipeline of generating the whole torso models, including implanted ICD electrode configurations. To begin with, segmentation of major organs, skin, and bones was performed using Simpleware (Version P-2019.09; Synopsys, Inc., Mountain View, USA). The hearts were segmented separately by firstly using an automatic tool (Siemens Axseg v4.11^24^), to obtain separate labels for left ventricular myocardium and individual blood pools of LV/RV, left/right atriums, and aorta. The walls of the RV, left/right atrium, and aorta were subsequently segmented manually in Simpleware, by dilating the blood pools to 3.5mm for the right ventricular wall and 2 mm for the left/right atrium and aorta, and the overlapping regions adjusted. In addition, the blood pools and walls of pulmonary veins and the SVC were also separately segmented. The whole segmentation process was similar to our previously reported approach.^25^

#### Representing hypertrophic and dilated structural variants

The five model hearts created above were initially assessed for pathological structural differences, based on measured dimensions reported in the literature:^26^ In doing so, two of them were found to have hypertrophic cardiomyopathy (HCM). In order to expand our cohort, heart geometries were modified to produce three structurally different hearts for each case, being healthy heart, along with HCM and dilated cardiomyopathy (DCM) variants, using a similar approach as Plancke *et al*.^23^ Specifically, HCM was represented by dilating the LV free wall of the healthy heart homogeneously into the LV blood pool; DCM was replicated by radially dilating the LV wall of the healthy heart along the LV’s long axis, with attenuated dilation applied at the apex and base of the LV. For the two original HCM hearts, the LV blood pool was dilated gradually into the LV wall, thinning the LV myocardial wall homogenously, until the healthy heart ventricular wall thickness (10–14 mm) was achieved. Dilated cardiomyopathy hearts were then generated from this synthetic ‘healthy’ heart. In summary, the key dimensions of HCM and DCM hearts, such as the LV end-diastolic diameters (LVEDD) and the ventricular wall thicknesses as compared against the healthy heart, are shown in *Table 1*, which are comparable to the literature.^23^

**Table 1:** Left ventricle end-diastolic diameters and the ventricular wall thicknesses of healthy, DCM, and HCM hearts

	LVEDD/mm		Ventricular wall thickness/mm	
Structure	Healthy	DCM	Healthy	HCM
Model 1	48	68	13.5	19.5
Model 2	45	65	11.7	20
Model 3	42	62	12.5	22
Model 4	46	65	12	18
Model 5	54	74	11	17

DCM, dilated cardiomyopathy; HCM, hypertrophic cardiomyopathy; LVEDD, left ventricle end-diastolic diameter.

#### Representing ischaemic cardiomyopathy

To replicate ischaemic cardiomyopathy, five different infarct scars reconstructed from late gadolinium–enhanced MRI of infarcted porcine hearts^27–29^ were randomly selected and mapped to the five healthy hearts. The scar locations were selected to represent a variety of typical perfusion territories: left anterior descending artery, left circumflex artery, and right coronary artery, as shown in Supplementary material online, *Figure S1A*. Scars were mapped using the universal ventricular coordinates.^30^ Overall, the four heart variants of each torso were then combined with the corresponding torso segmentations, which provided 20 whole torso models in total.

#### Implantable cardioverter defibrillator electrode representation

The virtual coils/cans for each ICD configuration were subsequently implanted into the torso models. *Figure 1B* shows schematic representations of the different ICD configurations used to virtually apply defibrillation shocks. Standard transvenous ICD configurations were modelled, including an RV shocking electrode (cylinder 2 mm diameter and 8 cm length) and a pectoral can (6 cm diameter and 1.3 cm length similar to Plancke *et al.*^23^) placed in the upper-left chest just below the clavicle (‘left-sided can’). Modifications of standard configurations included moving the can towards the upper-right chest (‘right-sided can’) and moving the RV coil up towards the mid-septum (‘RV septal coil’).

To thoroughly assess the effect of additional clinically available coils on DFT, an additional SVC coil (2 mm diameter and 8 cm length^23,20^) and CS coil (cylinder 2 mm diameter and 4 cm length^23,20^), similar to current clinically used leads in CRT-D, were also included.

#### Finite element model creation

Following the placement of all leads and cans, finite element meshes were created for the 20 models using Simpleware, producing meshes with average edge lengths of 800±50μm for LV and RV free walls, 2±1mm for organs and other non-myocardium regions, and 2±1.5mm for all coils and cans. Note that the coils and cans were regions embedded within the non-myocardium regions; thus, their surfaces were meshed smoothly without any sharp edges. Realistic cardiac fibre architecture for all 20 models was reconstructed using a rule-based approach^31^ to reproduce the anisotropic conduction within ventricles.

### Electrophysical model of cardiac tissue

The Cardiac Arrhythmia Research Package^32^ (https://carpentry.medunigraz.at/) was used to solve Laplace’s equation which describes the electrical potential distribution through the torso between the electrodes:


(1) $$\nabla \cdot \left(\sum \nabla V_{e}\right)=0,$$

where σ are the conductivities for different regions and *V*_e_ is the extracellular potential throughout all domains. At the boundaries of the torso, no flux conditions are imposed. Similar to our previous works,^23,33^ the organs and bath are considered as resistive conductors with homogeneous constant conductivities, as shown in *Table 2*. Myocardial anisotropic conductivities within the intra- and extra-cellular spaces were $\sigma_{\mathrm{il}}=0.174$ and $\sigma_{\mathrm{el}}=0.625\;S/m$ along the fibre direction and $\sigma_{\mathrm{it}}=0.019$ and $\sigma_{\mathrm{et}}=0.236\;S/m$^34^ transverse to the fibres.

**Table 2:** Conductivities for all regions in torso models, except for LV and RV myocardium

Regions	Conductivity (S/m)
Inner body (bath)	0.24725
Skin	0.117
Bones	0.05
Kidneys	0.1667
Liver	0.1667
Stomach	0.1
Spleen	0.1
Lung	0.0714
Left atrial wall	0.25
Right atrial wall	0.25
Blood pools and cardiac valves	0.6667

Shocks were simulated by setting the extracellular potential of the shocking coils and the ground to dissimilar values. For each scenario of the ICD configuration, the other cans/leads were effectively ‘removed’, i.e. set to have the same electrical properties as the surrounding regions.

### Data analysis

In order to quantitatively evaluate the impact of different ICD configurations, DFT was computed from the extracellular potential throughout the ventricular myocardium in all model configurations, following the application of a 10 V shock. Briefly, the DFT was then found by linearly scaling the applied simulation voltage to achieve the threshold, itself defined as the voltage level at which 95% of the ventricular myocardium achieves $∥\nabla V_{e}∥>5\;V/cm$. Such a criterion originates from the concept of ‘critical mass’ for the survival of fibrillatory wavefronts originating out of the seminal preclinical works^35,36^ and extensively used in prior simulation studies.^6,15–17,23^ Importantly, this exact methodology for DFT computation has recently been directly validated in a series of combined simulation and pre-clinical^21^ and clinical studies.^22^ The DFT energy was then calculated from the DFT voltage based on capacitive discharge^23,37^ by $\mathrm{Energy}=\frac{CV^{2}}{2}$, where the capacitance *C* is 100μF and *V* is the required DFT voltage. Mean electric field (E-field) (defined as $∥\nabla V_{e}∥$, gradient of extracellular potential) within the myocardium was also computed by applying a constant 10 V defibrillation shock to all models.

The impedance of different ICD settings was calculated via Ohm’s law: $R=\frac{V}{I_{\mathrm{total}}}$, where *V* is the voltage difference between the shocking electrode and the cans/grounds and $I_{\mathrm{total}}$ is the total current passing into the can/grounds, computed across each surface triangle of the electrodes (and summed). Specifically, the current injected (or passing) across each surface triangle was calculated by multiplying the surface area with the current density injected in the surface, itself derived from the mean electric field of three nodes of the triangle multiplied by the conductivity of the tissue surrounding the electrode, i.e. the bath or blood pool.

The efficacy between different pairs of ICD configurations was compared with Wilcoxon signed-rank tests. Continuous variables are presented as DFT, mean E-field, and impedance. The statistical significance for all tests was *P* < 0.05. The results below are reported as median and inter-quartile range, unless specified.

## Results

### Comparison between left-sided and right-sided can configurations

**Figure 2.**
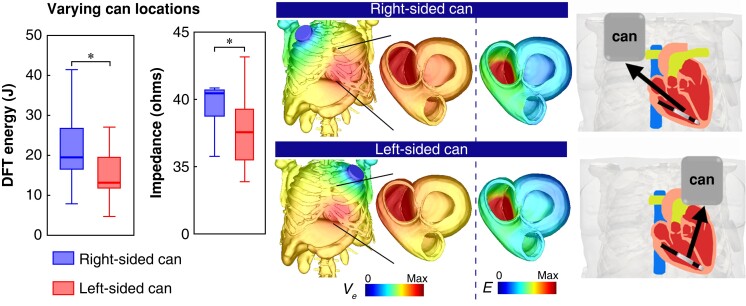
Comparison between Left-sided can (pink bar) with Right-sided can (black bar) configurations (with RV apical coil) for both DFT Energy (J) and electrical impedance (Ω) in Tukey boxplots (*n*=20). On the right shows the extracellular potential Ve (V) distribution across the torso model (transparent outer surfaces) and on both ventricles, as well as the gradient of Ve (electric field, E (V/cm)) across both ventricles.

*Figure 2* (left) confirms that the DFT when using a right-sided can was significantly higher than using a left-sided can [19.5 (16.4, 27.1) J vs. 13.3 (11.7, 19.9) J, *P* < 0.001], as expected. This is also consistent with the trend seen in the mean E-field (see Supplementary material online, *Figure S2A*), which shows that the right-sided can configuration has a significantly smaller E-field strength than the left-sided can [0.027 (0.026, 0.03) vs. 0.033 (0.031, 0.037) V/mm, *P* < 0.001]. In addition, as shown in *Figure 2* (centre), the impedance of the right-sided can is also significantly larger than the left-sided can [40.5 (38.7, 40.8) Ω vs. 37.6 (35.4, 39.3) Ω, *P* < 0.001].

### Mitigating increased defibrillation threshold of right-sided can configuration through the use of additional coils

**Figure 3.**
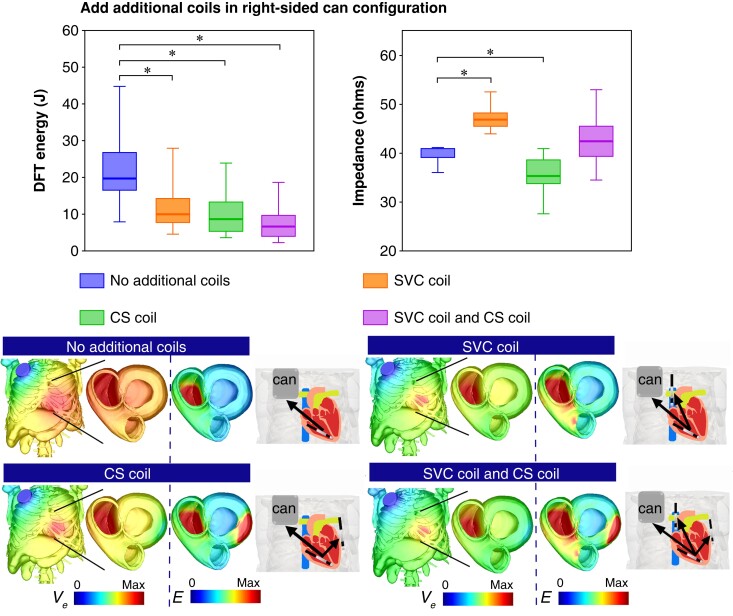
Comparison of DFT Energy (J) and electrical impedance (Ω ) with ICD configurations involving an RV apical coil and right-sided can with additional “SVC coil”, “CS coil” and “both SVC and CS coils” in Tukey boxplots (*n*=20). Lower images show the extracellular potential Ve (V) distribution for each configuration across the torso and on both ventricles, along with the gradient of Ve (electric field, E (V/cm)) across both ventricles.

As the right-sided can configuration results in a much higher DFT than the conventional (left-sided can) configuration, we quantified how the addition of further grounding coils may optimize its defibrillation efficacy, as shown in *Figure 3*. Here, the DFT decreased significantly from 19.5 (16.4, 27.1) J (no additional coils) to 10 (7.5, 14.6) J with an additional SVC coil, to 8.7 (5.2, 13.5) J with an additional CS coil, and to just 6.6 (3.9, 9.9) J with both coils added (all *P* < 0.001). This is consistent with the trend shown by the mean E-field [0.027 (0.026, 0.03) vs. 0.04 (0.037, 0.043), 0.04 (0.037, 0.046), and 0.047 (0.045, 0.052) V/mm, *P* < 0.001, respectively] (see Supplementary material online, *Figure S2B*). In the case of impedance (*Figure 3*), comparing to the right-sided can configuration, adding an SVC coil significantly increases impedance [40.5 (38.7, 40.8) Ω vs. 46.8 (45.2, 48.1) Ω, *P* < 0.001], but conversely adding a CS coil decreases impedance [35.2 (33.4, 38.6) Ω, *P* < 0.001], while adding both coils slightly increases impedance [42.2 (38.9, 45.4) Ω, *P* = 0.294].

### Assessing efficacy for mid-septal right ventricular coil placements

**Figure 4.**
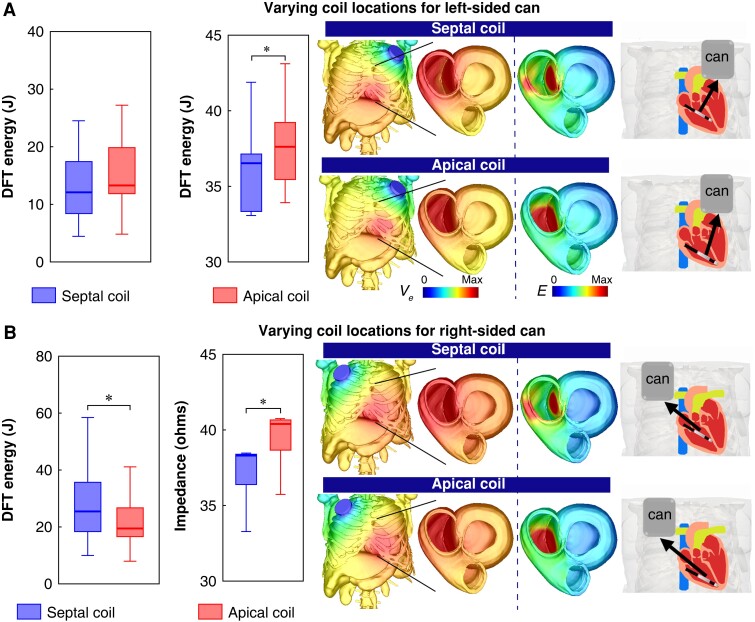
Comparison of DFT Energy (J) and electrical impedance (Ω ) with ICD configurations involving an RV septal coil and RV apical coil on in combination with (A) right-sided can and (B) left-sided can in Tukey boxplots (n=20). Images on the right show the extracellular potential Ve (V) distribution for each configuration across the torso and on both ventricles, along with the gradient of Ve (electric field, E (V/cm)) across both ventricles.

Positioning the RV coil towards the mid-septum may often be necessary to avoid regions of apical scar. *Figure 4A* compares the defibrillation efficacies of using an RV septal coil with an RV apical coil in both cases of using left-sided or right-sided cans. As shown, when used in conjunction with a left-sided can, the DFT of using an RV septal coil is not significantly different from using an RV apical coil [13.3 (11.7, 19.9) J vs. 12.1 (8.1, 17.6) J, *P* = 0.099]. Interestingly, the mean E-field when using an RV apical coil is significantly larger than using an RV septal coil [0.027 (0.026, 0.03) vs. 0.024 (0.02, 0.027) V/mm, *P* = 0.002] (see Supplementary material online, *Figure S3A*). In the case of impedance, when using an RV apical coil, impedance remains significantly larger than for an RV septal coil when using a left-sided can [37.6 (35.4, 39.3) Ω vs. 36.5 (33.2, 37.2) Ω, *P* < 0.001].

When using a right-sided can, as shown in *Figure 4B*, the DFTs are significantly higher when using an RV septal coil compared to an RV apical coil [26.7 (18.1, 36.1) J vs. 19.5 (16.4, 27.1) J, *P* < 0.001]. However, the impedance of the right-sided can configuration with an RV septal coil is also significantly smaller than when combined with an RV apical coil configuration [38.3 (36.4, 38.5) Ω vs. 40.5 (38.7, 40.8) Ω, *P* < 0.001]. Similar to the trend of DFTs, the mean E-field when using an RV septal coil is significantly smaller than when using an RV apical coil [0.024 (0.027, 0.02) vs. 0.027 (0.026, 0.03), *P* = 0.002] (see Supplementary material online, *Figure S3B*).

### Optimizing right ventricular septal coil to right-sided can configuration through additional extra coils

**Figure 5.**
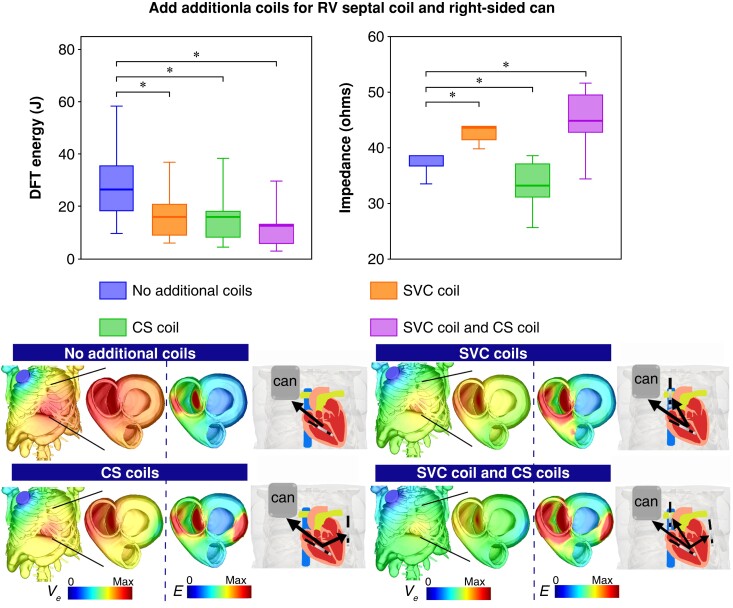
Comparison of DFT Energy (J) and electrical impedance (Ω) with ICD configurations involving an RV septal coil and Right-sided can with additional SVC coil, CS coil and both SVC and CS coils in Tukey boxplots (*n*=20). Lower images show the extracellular potential Ve (V) distribution for each configuration across the torso and on both ventricles, along with the gradient of Ve (electric field, E(V/cm)) across both ventricles.

As shown above, having to implant the RV coil towards the mid-septum results in a detrimental increase in DFT when a right-sided can is also necessitated. Therefore, we investigated how additional ground electrodes may help mitigate this increase. *Figure 5* shows that, when using an RV septal coil and right-sided can configuration, DFT is significantly reduced through the addition of an SVC coil, CS coil, and both SVC and CS coils [26.7 (18.1, 36.1) J (no additional coils) vs. 16.1 (8.9, 20.9) J, 16.1 (8.1, 18.5) J, and 12.1 (5.7, 13.5) J, all *P* < 0.001, respectively]. This is also consistent with the trend in mean E-field [0.024 (0.027, 0.02) vs. 0.036 (0.03, 0.039), 0.036 (0.034, 0.041), and 0.046 (0.04, 0.048) V/mm, *P* < 0.001] (see Supplementary material online, *Figure S3C*). As shown in *Figure 5*, impedance increases significantly with adding an SVC coil and adding both coils [38.3 (36.4, 38.5) Ω vs. 43.1 (41.1, 43.8) Ω and 44.7 (42.4, 49.4) Ω, both *P* < 0.001, respectively], but conversely decreases with adding a CS coil [32.9 (30.8, 37) Ω, *P* < 0.001].

### Defibrillation threshold assessment on different cardiac pathologies

**Figure 6.**
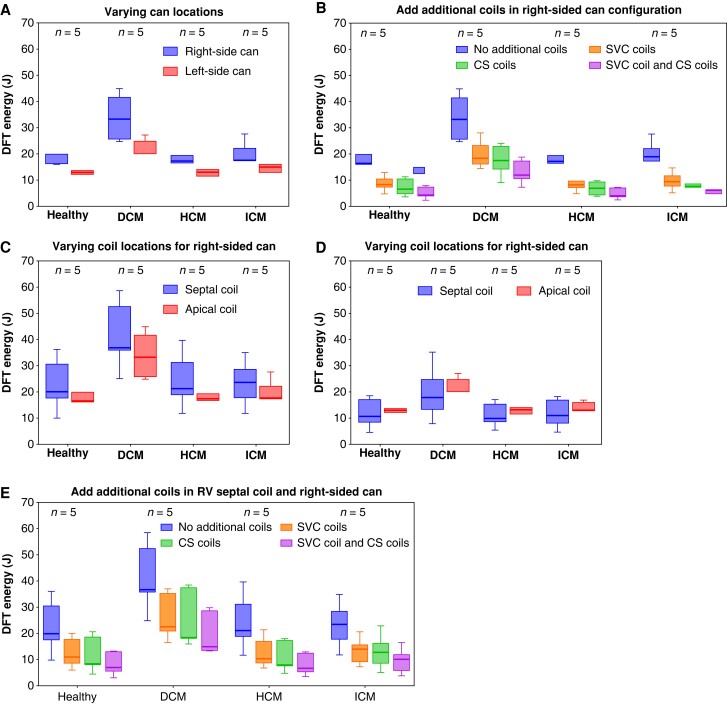
Comparison of DFT Energy (J) with different cardiac pathological variants separated into healthy, DCM, HCM and ICMs using ICD configurations as in Figure 2 (*A*), 3 (*B*), 4 (*C*, *D*), 5 (*E*).

Given the inclusion of different cardiac pathologies within our torso cohort, we further analysed our data to look for any trends with respect to DFT changes between configurations within HCM, DCM, ICM, and healthy variants. *Figure 6* presents the DFTs in different pathological cardiac variants (HCM, DCM, and ICM) along with healthy hearts, using all ICD configurations reported above. Here, we see a consistent increase when moving the can to the right in all variants (consistent with the overall trend in *Figure 2*), with a noticeably stronger trend in DCM variants (median: 33.2 J vs. 20.1 J), compared to the others (median: 16.3–17.4 J vs. 12.88–13.1 J) albeit non-significant (*Figure 6A*). As in *Figure 3*, adding both SVC and CS coils is seen to reduce DFT in the right-sided can configuration the most in all variants, while this trend appears to be more distinct in HCM variants and healthy hearts (median: 17.2 vs. 4 J and 16.3 vs. 4.22 J) than ICM and DCM variants (median: 17.4 vs. 6 J and 33.2 vs. 12 J) (*Figure 6B*). Moving the RV coil to septal locations with a right-sided can increases DFTs in all variants, while with a left-sided can, there appears to be a trend for DFT to be decreased slightly (*Figure 6C* and *D*). Similar to *Figure 5*, adding both SVC and CS coils reduce DFTs in all cardiac variants. Further subanalyses of DFT changes based on models with different scar locations within the five ICM torsos are shown in Supplementary material online, *Figure S1B–F*.

## Discussion

In this study, we utilized an *in silico* cohort of whole torso models to quantitatively assess the change in defibrillation efficacy when using a right-sided can and/or a RV septal coil. The flexible and controlled nature of our *in silico* approach allowed us to quantitatively determine how any reductions in efficacy (increased DFTs) may be mitigated in each scenario, through the addition of clinically available coils, such as those placed in the SVC and/or CS. Our main findings are:

1. Right-sided can configurations significantly increase DFT compared to conventional left-sided cans due to a significant reduction of the mean E-field throughout the ventricles.
2. Septal RV coil placement did not significantly affect DFT when using a left-sided can but did significantly increase DFT when using a right-sided can compared to the apical coil location.
3. Defibrillation efficacy of the right-sided can configuration (with either apical or septal coil) can be improved significantly through additional ground coils in the SVC and/or CS, requiring approximately one-third of the energy compared to the original configurations with no additional coils.
4. The mean E-field is often correlated with DFT, as is the impedance, but to a lesser extent.

### Quantifying defibrillation threshold increase when a right-sided can is required

In certain patient groups, for example those having pocket infections from previously implanted devices, those with occlusion of left central veins, presence of permanent left catheters (e.g. dialysis), prior surgery on the left chest, or preferences due to left-handedness, a can is often implanted to the right chest. This patient group has been estimated to comprise 2–12% of all ICD implants.^11,38^ Moving the can to the right chest increases its distance from the shocking coil and also varies the direction of the shock vector such that it passes through less of the LV myocardium. Our simulations showed the right-sided can configuration results in approximately a 50% increase of DFT and also 8% increase in impedance, driven by a smaller E-field strength across the LV for driving the change of membrane potential required to defibrillate. Our findings are consistent with both previous clinical and simulation studies^6,11–13,20^ that have shown a more proximal can yields a lower DFT. Although DFT testing is not commonplace with conventional left-sided implants, our data suggest that the significant differences seen in DFT for right-sided implants may warrant DFT testing whenever a right-sided implant is required.

### Mid-septal right ventricular coil location increases defibrillation threshold for right-sided can configurations

In patients with a known apical scar, the RV coil may need to be implanted further from the apex towards the mid-septum to ensure adequate capture of pacing from the RV lead tip (during ATP, for example). Our study shows that, compared to a standard apical coil location, having a mid-septal coil (in the standard left-sided can configuration) shows a non-significant lowering of DFT (*Figure 4A*), accompanied by a small (significant) decrease in impedance. However, a more noticeable decrease in the mean E-field was seen for a mid-septal coil (see Supplementary material online, *Figure S3A*), particularly observed in its distribution within the RV (*Figure 4A*). These findings are in agreement with previous studies which also reported a similar trend of a septal coil decreasing DFT,^16,17,39^ albeit with different shock vectors (SVC coil as the only cathode or both can and SVC coil as cathodes).

In contrast, in the context of a right-sided can, moving the RV coil towards the mid-septum significantly increased DFT (∼30%, *Figure 4B*) as well as impedance. This increase in DFT is driven by the septal coil configuration resulting in less volume of myocardium having high E-field across both ventricles.

Alternatively viewed, if an apical RV coil implantation is not possible (due to scarring), necessitating a mid-septal implantation, our findings suggest that an additional decision to implant the can on the right side would result in approximately a 100% increase in DFT (50% increase if apical RV coil is possible). This large increase is due to the fact that the shock vector between the right-sided can and a septal RV coil passes through the smallest amount of LV myocardium, as observed reduction of E-field strength throughout both ventricles (*Figure 4B*), requiring more energy for defibrillation. Thus, if at all possible, in the context of a necessitated mid-septal RV coil implantation, a left-sided location should always be preferred to avoid unwanted large increases in required DFTs.

The inclusion of a subset of modified models containing scars also allowed us to quantify these data in the more realistic scenario of ischaemic cardiomyopathy patients. In these cases, our results suggested a slight trend in the scar acting to increase the DFT. While this is plausible, as mechanistically scar introduces a lower conductive region which increases the E-field strength across the infarct, while decreasing it within the myocardium, increasing DFT, the overall increases seen in scarred models were relatively minor (<10 J) and warrants further investigation with greater sample size.

### Additional superior vena cava and coronary sinus coils improve defibrillation efficacy of right-sided can configuration with/without a right ventricular septal coil

For decades, there has been continued debate as to whether additional coils (such as an SVC coil in a dual-coil system) improve defibrillation efficacy and reduce mortality. Multiple clinical and simulation studies have shown that dual-coil systems have lower DFT than single-coil systems,^6,7,15^ while others show more mixed results,^3^ particularly in the context of right-sided can configurations.^11^

Our simulations showed that additional coils indeed significantly improve defibrillation efficacy for only an SVC coil, only a CS coil, or the addition of both coils, but to different extents. For the right-sided can configuration (*Figure 3*), the addition of either SVC, CS, or both coils significantly reduces DFT (by up to 70% to just 6.6 J for both coils). Importantly, this attenuation of DFT acts to offset the previous increase seen in the right-sided can configuration, such that it is now below the DFT of the left-sided can with no additional coils.

Our detailed computational analysis of the E-field distributions in *Figure 3* showed that adding an SVC coil results in more RV tissue with higher E-field, whereas more LV tissue has higher E-field when adding a CS coil, while adding both coils results in higher E-field across both ventricles.

In the context of an RV septal coil and right-sided can configuration, additional coils once again reduced DFT significantly (compared to no additional coils), again largely mitigating the detrimental effects of these unconventional can and RV coil placements. Analysis of the E-field distribution showed that adding both coils resulted in the highest E-field across both ventricles compared with only SVC or CS coil configuration (*Figure 5*), therefore causing the largest reduction in DFT.

Furthermore, it is interesting to notice that despite using apical/septal coils, adding only the CS coil resulted in the lowest impedance compared with the other two additional coil configurations, as the CS coil introduced a shocking vector targeting the LV and also formed a shorter current path. However, lower impedance does not necessarily drive higher defibrillation efficacy.^40^

### Safety considerations of additional coils

Implantation of extra coils may be associated with increased risks of lead-related complications, especially in the longer term, for example during lead extraction.^6^ Consequently, the dual-coil system is less extensively used nowadays,^41^ only in specific cases such as patients with HCM which may require higher energies for defibrillation.^42^ Placement of leads in the CS is now routinely used to optimize pacing sequences in CRT devices, but is also emerging as a novel approach for defibrillation,^43,44^ which also may be particularly useful for patients with a higher DFT.^9^

Unfortunately, shocking coils are known to have a higher tendency to adhere to veins than standard pacing leads, which may cause safety concerns, particularly in the case of required extraction. However, we have previously reported on a patient who had a shocking coil present in their CS for 115 months, which was later successfully extracted using laser extraction.^45^ Although not without risk, there is important clinical evidence to suggest that the placement of a shocking coil within the CS can be useful to achieve a lower DFT in specific problematic cases where DFTs are unacceptably high,^8,9^ which we have also shown in cases at our institution.^46^ The findings in this work may justify the consideration of corresponding additional coils in patients who require right-sided can implants, with potentially only an additional CS coil suggested in the additional case of an enforced mid-septal RV coil.

### Clinical implications

Ultimately, the optimal ICD configuration has to take into account not only DFT but also impedance (which impacts current drain and battery performance), as well as the effect of the shock on surrounding tissues and organs, which represents important considerations in ICD device design. Although implantation of more coils may increase the risks of complications and may be also difficult for extraction, novel lead designs are emerging that show the potential to reduce lead-related complications, i.e. reducing fibrotic adhesions on the lead surface.^47^ In addition, other lead-less and bioresorbable technology is emerging which may eliminate these complications and still be able to replicate similar shock vectors as shown here.^48,49^

### Limitations

Unfortunately, due to a lack of specific DFT testing in routine clinical practice, direct validation of the DFT predictions from our modelling approach was not possible in this specific context. However, we emphasize that the computational methodology employed here for DFT estimation is identical to that used in a number of recent stimulation studies which have successfully directly validated this approach in pre-clinical^21^ and also clinical data^22^ for ICD modelling, as well as in a recent subcutaneous ICD optimization modelling study.^19^ Unfortunately, although we have presented data here which show potentially interesting trends, we did not have sufficient model numbers in each heart variant category to comprehensively investigate specific differences in DFT or configuration dependence between different cardiac pathologies and/or scar locations with adequate statistical power and leave this as a line of interesting further investigation with larger cohorts.

## Conclusions

Implanting a right-sided generator may increase DFT by 50% compared to left-sided can placement. This increase may be mitigated by adding additional coils in the SVC and CS (as extra grounds), reducing required defibrillation energies to be similar to standard left-sided single-coil ranges, with coils in both SVC and CS together bringing the lowest DFT. Right ventricular coil septal placement may reduce DFT slightly when using a left-sided can; however, it significantly increases DFT when using a right-sided can. This suggests, in cases where right-sided can placement is mandated, a septal lead position should be avoided. If septal positioning is necessary, the DFT may be significantly lowered by the addition of both SVC and CS coils.

## Supplementary Material

euad146_Supplementary_Data

## Data Availability

The computational torso models developed here are available to the public via online data repository: https://doi.org/10.5281/zenodo.7253863.

## References

[1] Haqqani HM , Mond HG. The implantable cardioverter-defibrillator lead: principles, progress, and promises: principles. Pacing Clin Electrophysiol 2009;32:1336–53. 19796348 10.1111/j.1540-8159.2009.02492.x

[2] Sunderland N , Kaura A, Murgatroyd F, Dhillon P, Scott PA. Outcomes with single-coil versus dual-coil implantable cardioverter defibrillators: a meta-analysis. Europace 2018;20:e21–9. 28339860 10.1093/europace/euw438

[3] Larsen JM , Hjortshøj SP, Nielsen JC, Johansen JB, Petersen HH, Haarbo J et al Single-coil and dual-coil defibrillator leads and association with clinical outcomes in a complete Danish nationwide ICD cohort. Heart Rhythm 2016;13:706–12. 26593333 10.1016/j.hrthm.2015.11.034

[4] Aoukar PS , Poole JE, Johnson GW, Jill Anderson R, Hellkamp AS, Mark DB et al No benefit of a dual coil over a single coil ICD lead: evidence from the Sudden Cardiac Death in Heart Failure Trial. Heart Rhythm 2013;10:970–6. 23562699 10.1016/j.hrthm.2013.03.046

[5] Rinaldi CA , Simon RDB, Geelen P, Reek S, Baszko A, Kuehl M et al A randomized prospective study of single coil versus dual coil defibrillation in patients with ventricular arrhythmias undergoing implantable cardioverter defibrillator therapy. Pacing Clin Electrophysiol 2003;26:1684–90. 12877701 10.1046/j.1460-9592.2003.t01-1-00253.x

[6] Jolley M , Stinstra J, Pieper S, Macleod R, Brooks DH, Cecchin F et al A computer modeling tool for comparing novel ICD electrode orientations in children and adults. Heart Rhythm 2008;5:565–72. 18362024 10.1016/j.hrthm.2008.01.018 PMC2745086

[7] Neuzner J , Carlsson J. Dual-versus single-coil implantable defibrillator leads: review of the literature. Clin Res Cardiol 2012;101:239–45. 22231644 10.1007/s00392-011-0407-z

[8] Wilczek R , Świa̧tkowski M, Czepiel A, Sterliński M, Makowska E, Kułakowski P. Implantation of additional defibrillation lead into the coronary sinus: an effective method of decreasing defibrillation threshold. Kardiol Pol 2011;69:1308–9. 22219117

[9] Rodríguez-Mañero M , Kreidieh B, Ibarra-Cortez SH, Álvarez P, Schurmann P, Dave AS et al Coronary vein defibrillator coil placement in patients with high defibrillation thresholds. J Arrhythm 2019;35:79–85. 30805047 10.1002/joa3.12136 PMC6373648

[10] Alter P , Waldhans S, Plachta E, Moosdorf R, Grimm W. Complications of implantable cardioverter defibrillator therapy in 440 consecutive patients. Pacing Clin Electrophysiol. 2005;28:926–32. 16176531 10.1111/j.1540-8159.2005.00195.x

[11] Varma N , Efimov I. Right pectoral implantable cardioverter defibrillators: role of the proximal (SVC) coil. Pacing Clin Electrophysiol 2008;31:1025–35. 18684259 10.1111/j.1540-8159.2008.01130.x

[12] Varma N , Schaerf R, Kalbfleisch S, Pimentel R, Kroll MW, Oza A. Defibrillation thresholds with right pectoral implantable cardioverter defibrillators and impact of waveform tuning (the Tilt and Tune trial). Europace 2017;19:1810–7. 27986795 10.1093/europace/euw306

[13] Friedman PA , Rasmussen MJ, Grice S, Trusty J, Glikson M, Stanton MS. Defibrillation thresholds are increased by right-sided implantation of totally transvenous implantable cardioverter defibrillators. Pacing Clin Electrophysiol 1999;22:1186–92. 10461295 10.1111/j.1540-8159.1999.tb00599.x

[14] Lang DJ , Heil JE, Hahn SJ, Lindstrom CC, Derfus DL. Implantable cardioverter defibrillator lead technology: improved performance and lower defibrillation thresholds. Pacing Clin Electrophysiol 1995;18:548–59. 7777419 10.1111/j.1540-8159.1995.tb02565.x

[15] de Jongh AL , Entcheva EG, Replogle JA, Booker RS, Kenknight BH, Claydon FJ. Defibrillation efficacy of different electrode placements in a human thorax model. Pacing Clin Electrophysiol 1999;22:152–7. 9990621 10.1111/j.1540-8159.1999.tb00323.x

[16] Aguel F , Eason JC, Trayanova NA, Siekas G, Fishler MG. Impact of transvenous lead position on active-can ICD defibrillation: a computer simulation study. Pacing Clin Electrophysiol 1999;22:158–64. 9990622 10.1111/j.1540-8159.1999.tb00324.x

[17] Yang F , Patterson R. Optimal transvenous coil position on active-can single-coil ICD defibrillation efficacy: a simulation study. Ann Biomed Eng 2008;36:1659–67. 18688723 10.1007/s10439-008-9548-2

[18] Jolley M , Stinstra J, Tate J, Pieper S, Macleod R, Chu L et al Finite element modeling of subcutaneous implantable defibrillator electrodes in an adult torso. Heart Rhythm 2010;7:692–8. 20230927 10.1016/j.hrthm.2010.01.030 PMC3103844

[19] Heist EK , Belalcazar A, Stahl W, Brouwer TF, Knops RE. Determinants of subcutaneous implantable cardioverter-defibrillator efficacy: a computer modeling study. JACC Clin Electrophysiol 2017;3:405–14. 29759454 10.1016/j.jacep.2016.10.016

[20] Rantner LJ , Vadakkumpadan F, Spevak PJ, Crosson JE, Trayanova NA. Placement of implantable cardioverter-defibrillators in paediatric and congenital heart defect patients: a pipeline for model generation and simulation prediction of optimal configurations. J Physiol 2013;591:4321–34. 23798492 10.1113/jphysiol.2013.255109 PMC3779119

[21] Tate JD , Pilcher TA, Aras KK. Burton BM, Macleod RS. Validating defibrillation simulation in a human-shaped phantom. Heart Rhythm 2020;17:661–8. 31765807 10.1016/j.hrthm.2019.11.020 PMC7117986

[22] Tate J , Stinstra J, Pilcher T, Poursaid A, Jolley MA, Saarel E et al Measuring defibrillator surface potentials: the validation of a predictive defibrillation computer model. Comput Biol Med 2018;102:402–10. 30195579 10.1016/j.compbiomed.2018.08.025 PMC6221557

[23] Plancke AM , Connolly A, Gemmell PM, Neic A, Mcspadden LC, Whitaker J et al Generation of a cohort of whole-torso cardiac models for assessing the utility of a novel computed shock vector efficiency metric for ICD optimisation. Comput Biol Med 2019;112:103368. 10.1016/j.compbiomed.2019.103368 PMC6873640 31352217

[24] Zheng Y , Barbu A, Georgescu B, Scheuering M, Comaniciu D. Four-chamber heart modeling and automatic segmentation for 3-D cardiac CT volumes using marginal space learning and steerable features. IEEE Trans Med Imaging 2008;27:1668–81. 18955181 10.1109/TMI.2008.2004421

[25] Strocchi M , Augustin CM, Gsell MAF, Karabelas E, Neic A, Gillette K et al A publicly available virtual cohort of fourchamber heart meshes for cardiac electromechanics simulations. PLoS One 2020;15:1–26. 10.1371/journal.pone.0235145 PMC7319311 32589679

[26] Lang RM , Bierig M, Devereux R, Flachskampf F, Foster E, Pellikka P et al Recommendations for chamber quantification. Eur J Echocardiogr 2006;7:79–108. 16458610 10.1016/j.euje.2005.12.014

[27] Whitaker J , Neji R, Kim S, Connolly A, Aubriot T, Calvo JJ et al Late gadolinium enhancement cardiovascular magnetic resonance assessment of substrate for ventricular tachycardia with hemodynamic compromise. Front Cardiovasc Med 2021;8:744779. 34765656 10.3389/fcvm.2021.744779 PMC8576410

[28] Whitaker J , Neji R, Byrne N, Puyol-Antón E, Mukherjee RK, Williams SE et al Improved co-registration of ex-vivo and in-vivo cardiovascular magnetic resonance images using heart-specific flexible 3D printed acrylic scaffold combined with non-rigid registration. J Cardiovasc Magn Resonan 2019;21:1–15. 10.1186/s12968-019-0574-z PMC6785908 31597563

[29] Qian S , Connolly A, Mendonca-Costa C, Campos F, Rodero C, Whitaker J et al Optimization of anti-tachycardia pacing efficacy through scar-specific delivery and minimization of re-initiation: a virtual study on a cohort of infarcted porcine hearts. Europace 2022;44:1–10. 10.1093/europace/euac165 PMC9935023 36197749

[30] Bayer J , Prassl AJ, Pashaei A, Gomez JF, Frontera A, Neic A et al Universal ventricular coordinates: a generic framework for describing position within the heart and transferring data. Med Image Anal 2018;45:83–93. 29414438 10.1016/j.media.2018.01.005

[31] Bayer JD , Blake RC, Plank G, Trayanova NA. A novel rule-based algorithm for assigning myocardial fiber orientation to computational heart models. Ann Biomed Eng 2012;40:2243–54. 22648575 10.1007/s10439-012-0593-5 PMC3518842

[32] Vigmond EJ , Hughes M, Plank G, Leon LJ. Computational tools for modeling electrical activity in cardiac tissue. J Electrocardiol 2003;36:69–74. 14716595 10.1016/j.jelectrocard.2003.09.017

[33] Monaci S , Strocchi M, Rodero C, Gillette K, Whitaker J, Rajani R et al In-silico pace-mapping using a detailed whole torso model and implanted electronic device electrograms for more efficient ablation planning. Comput Biol Med 2020;125:104005. 10.1016/j.compbiomed.2020.104005 32971325

[34] Clerc L . Directional differences of impulse spread in trabecular muscle from mammalian heart. J Physiol 1976;255:335–46. 1255523 10.1113/jphysiol.1976.sp011283 PMC1309251

[35] Zipes DP , Fischer J, King RM, Nicoll A, Jolly WW. Termination of ventricular fibrillation in dogs by depolarizing a critical amount of myocardium. Am J Cardiol 1975;36:37–44. 1146696 10.1016/0002-9149(75)90865-6

[36] Zhou X , Daubert JP, Wolf PD, Smith WM, Ideker RE. Epicardial mapping of ventricular defibrillation with monophasic and biphasic shocks in dogs. Circ Res 1993;72:145–60. 8417837 10.1161/01.res.72.1.145

[37] Qian S , Connolly A, Mendonca-Costa C, Campos F, Williams SE, Whitaker J et al An in-silico assessment of efficacy of two novel intra-cardiac electrode configurations versus traditional anti-tachycardia pacing therapy for terminating sustained ventricular tachycardia. Comput Biol Med 2021;139:104987. 10.1016/j.compbiomed.2021.104987 PMC8669079 34741904

[38] Flaker GC , Tummala R, Wilson J. Comparison of right- and left-sided pectoral implantation parameters with the jewel active can cardiodefibrillator. Pacing Clin Electrophysiol. 1998;21:447–51 . 9507547 10.1111/j.1540-8159.1998.tb00070.x

[39] Winter J , Heil JE, Schumann C, Lin Y, Schannwell CM, Michel U et al Effect of implantable cardioverter/defibrillator lead placement in the right ventricle on defibrillation energy requirements. A combined experimental and clinical study. Eur J Cardiothorac Surg 1998;14:419–25. 9845149 10.1016/s1010-7940(98)00215-2

[40] Gold MR , Olsovsky MR, Pelini MA, Peters RW, Shorofsky SR. Comparison of single- and dual-coil active pectoral defibrillation lead systems. J Am Coll Cardiol 1998;31:1391–4. 9581739 10.1016/s0735-1097(98)00103-x

[41] Leshem E , Suleiman M, Laish-Farkash A, Konstantino Y, Glikson M, Barsheshet A et al Contemporary rates and outcomes of single- vs. dual-coil implantable cardioverter defibrillator lead implantation: data from the Israeli ICD Registry. Europace 2017;19:1485–92. 27702848 10.1093/europace/euw199 PMC5834150

[42] Kumar KR , Mandleywala SN, Madias C, Weinstock J, Rowin EJ, Maron BJ et al Single coil implantable cardioverter defibrillator leads in patients with hypertrophic cardiomyopathy. Am J Cardiol 2020;125:1896–900. 32305220 10.1016/j.amjcard.2020.03.011

[43] Janardhan AH , Li W, Fedorov VV, Yeung M, Wallendorf MJ, Schuessler RB et al A novel low-energy electrotherapy that terminates ventricular tachycardia with lower energy than a biphasic shock when antitachycardia pacing fails. J Am Coll Cardiol 2012;60:2393–8. 23141483 10.1016/j.jacc.2012.08.1001 PMC4573535

[44] Tonko JB , Rinaldi CA. Non-traditional implantable cardioverter-defibrillator configurations and insertion techniques: a review of contemporary options. Europace 2022;24:181–92. 34453529 10.1093/europace/euab178 PMC8824518

[45] Hamid S , Arujuna A, Rinaldi CA. A shocking lead in the coronary sinus. Europace 2009;11:833–4. 19363050 10.1093/europace/eup088

[46] Spurrell P , Gandhi M, Rinaldi CA. A biventricular ICD system with biventricular defibrillation. Pacing Clin Electrophysiol 2006;334:334–6. 10.1111/j.1540-8159.2006.00344.x 16606405

[47] di Cori A , Bongiorni MG, Zucchelli G, Segreti L, Viani S, Paperini L et al Transvenous extraction performance of expanded polytetrafluoroethylene covered ICD leads in comparison to traditional ICD leads in humans. Pacing Clin Electrophysiol 2010;33:1376–81. 20735715 10.1111/j.1540-8159.2010.02879.x

[48] de Maria E , Ziacchi M, Diemberger I, Biffi M. Leadless left ventricular endocardial pacing: a real alternative or a luxury for a few? Cardiovasc Diagn Ther 2018;8:530–3. 30214871 10.21037/cdt.2018.03.08 PMC6129828

[49] Choi YS , Yin RT, Pfenniger A, Koo J, Avila R, Benjamin Lee K et al Fully implantable and bioresorbable cardiac pacemakers without leads or batteries. Nat Biotechnol 2021;39:1228–38. 34183859 10.1038/s41587-021-00948-x PMC9270064

