# Supplementary material for: Additional coils mitigate elevated defibrillation threshold in right-sided implantable cardioverter defibrillator generator placement: a simulation study
Source: Europace. 2023 Jun 14;25(6):euad146. doi: 10.1093/europace/euad146 (PMC10265967; doi:10.1093/europace/euad146)
Supplement: euad146_Supplementary_Data [file euad146_supplementary_data.pdf]

Additional coils mitigate elevated defibrillation threshold in right-sided ICD generator placement: a simulation study

Shuang Qian, PhD,* Sofia Monaci, PhD,* Caroline Mendonca-Costa, PhD,* Fernando Campos, PhD,* Philip Gemmell, PhD,* Hassan A. Zaidi, MSc,* Ronak Rajani, MD, FESC, FRCP, FSCCT,§ John Whitaker, MD, PhD,*§ Christopher A. Rinaldi, MD, FRCP, FHRS,*§ Martin J. Bishop, PhD*

Supplement material

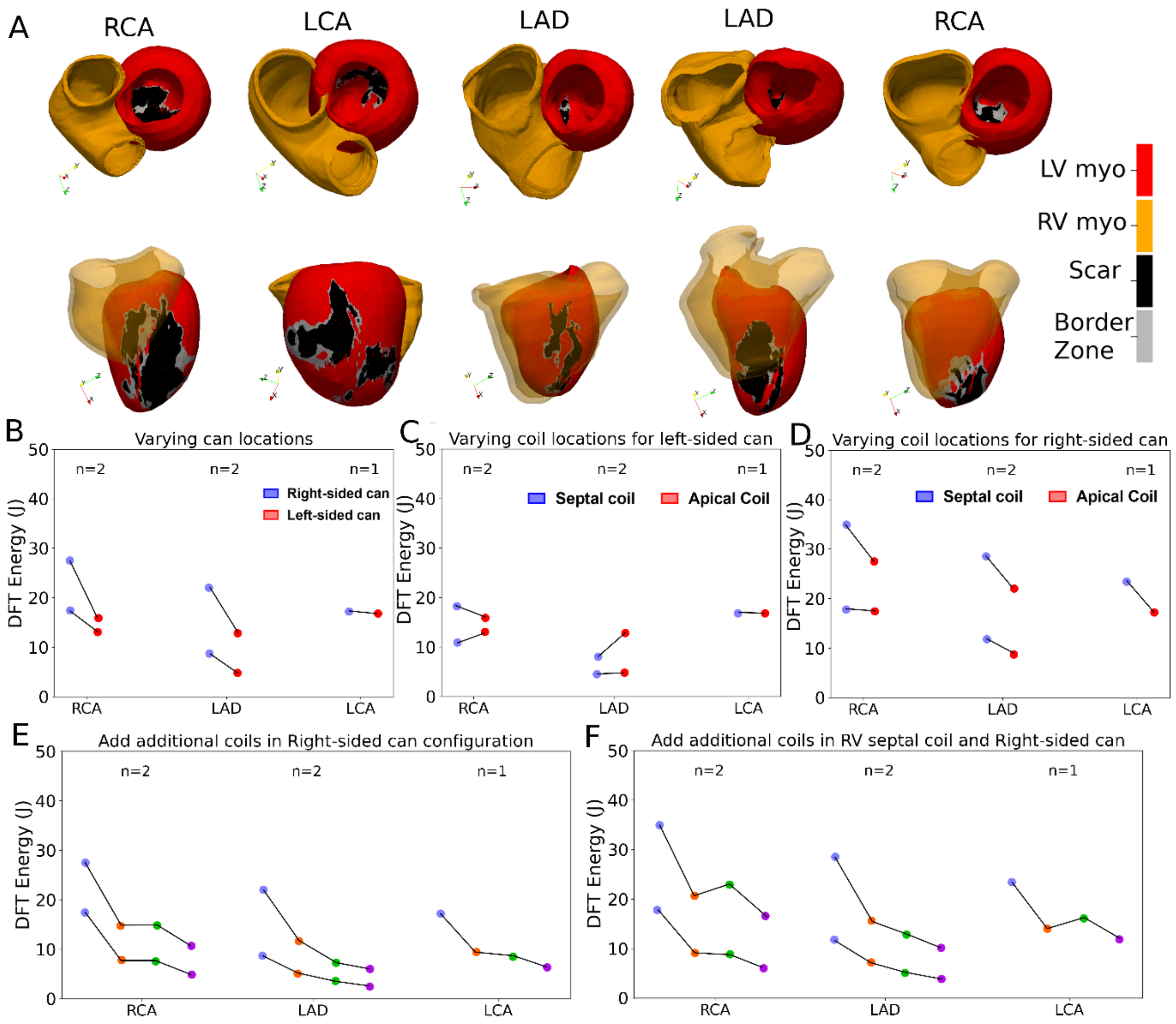

Supplemental Figure 1: A. Mapped infarct scars in five healthy hearts in perfusion territories of RCA, LCA and LAD. B-F: Comparison of DFT Energy (J) with scar patterns as RCA, LCA and LAD using ICD configurations as in Figure 2-5.

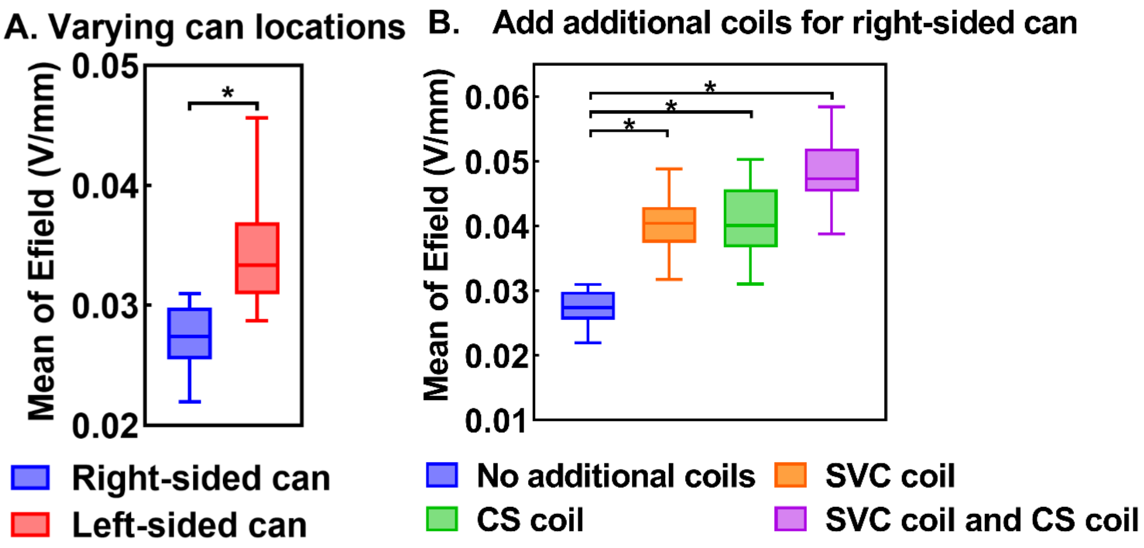

Supplemental Figure 2: Mean electric field for varying can locations. A. Comparison of shocking between shocking electrode at RV apex and left-sided can) (pink bar) with Right-sided can configuration (black bar) in Tukey boxplots (n=20). B. Comparison of shocking between Right-sided can configuration (black bar) with adding "SVC coil", "CS coil" and "both SVC and CS coils" in Tukey boxplots (n=20).

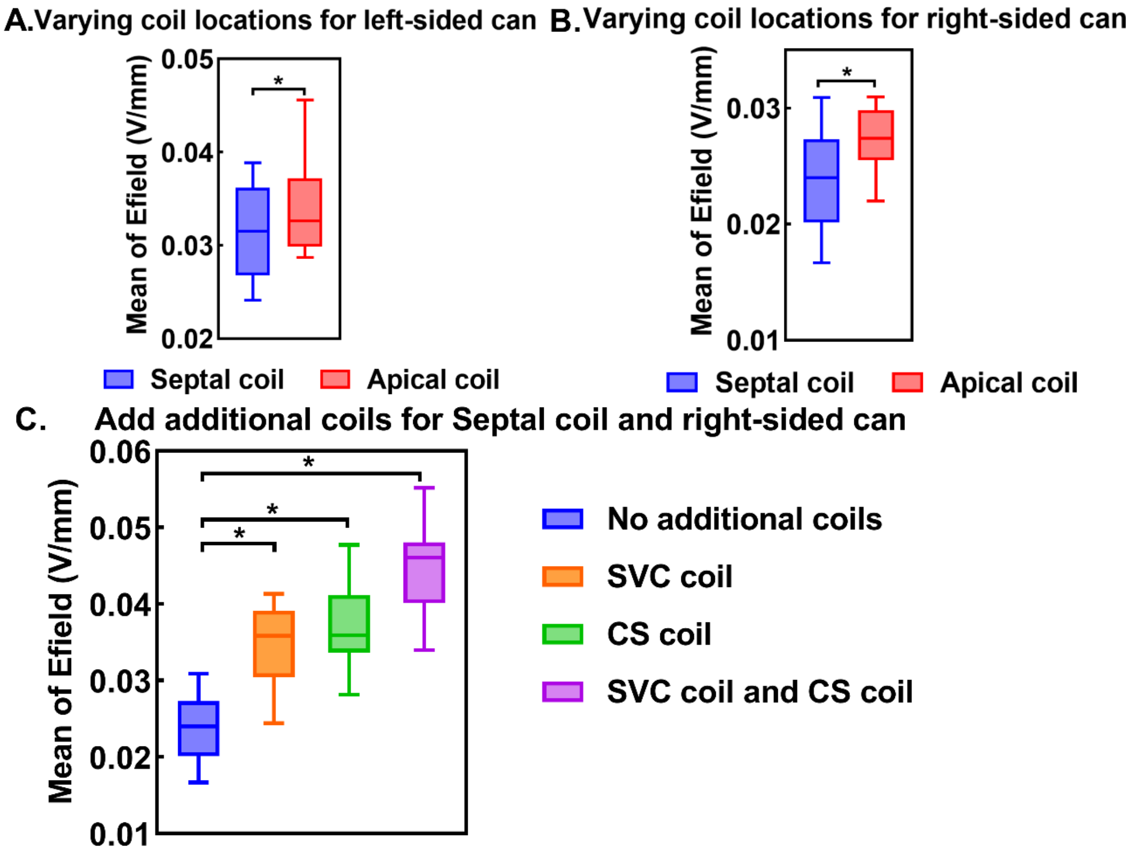

Supplemental Figure 3: Mean electric field for varying can locations. Comparison between RV septal coil and apical coil with using (A) right-sided can and (B) left-sided can in Tukey boxplots (n=20). (C) comparison of shocking between RV septal coil and right-sided can comparing with adding "SVC coil", "CS coil" and "add both SVC and CS coils" in Tukey boxplots (n=20).
